# Use of RE-AIM to develop a multi-media facilitation tool for the patient-centered medical home

**DOI:** 10.1186/1748-5908-6-118

**Published:** 2011-10-21

**Authors:** Russell E Glasgow, Perry Dickinson, Lawrence Fisher, Steve Christiansen, Deborah J Toobert, Bruce G Bender, L Miriam Dickinson, Bonnie Jortberg, Paul A Estabrooks

**Affiliations:** 1 Division of Cancer Control and Population Sciences, National Cancer Institute, 6130 Executive Blvd., Room 6144, Rockville, MD 20852, USA; 2 University of Colorado School of Medicine, 12631 East 17th Avenue, Aurora, CO 80045, USA; 3 Department of Family and Community Medicine, Diabetes Center, University of California, San Francisco, Parnassus Heights, Box 0900, 500 Parnassus Avenue, MU3E, San Francisco, CA 94143-0900, USA; 4 Intervision Media, 261 E.12th Avenue, Eugene, OR 97401,USA; 5 Oregon Research Institute, 1715 Franklin Blvd, Eugene, OR 97403, USA; 6 Division of Pediatric Behavioral Health, National Jewish Health, 1400 Jackson Street, Denver, CO 80206, USA; 7 Virginia Tech, Department of Human Nutrition, Foods and Exercise, VT Riverside, 1 Riverside Circle, SW Roanoke, VA 24016, USA

## Abstract

**Background:**

Much has been written about how the medical home model can enhance patient-centeredness, care continuity, and follow-up, but few comprehensive aids or resources exist to help practices accomplish these aims. The complexity of primary care can overwhelm those concerned with quality improvement.

**Methods:**

The RE-AIM planning and evaluation model was used to develop a multimedia, multiple-health behavior tool with psychosocial assessment and feedback features to facilitate and guide patient-centered communication, care, and follow-up related to prevention and self-management of the most common adult chronic illnesses seen in primary care.

**Results:**

The *Connection to Health *Patient Self-Management System, a web-based patient assessment and support resource, was developed using the RE-AIM factors of reach (*e.g*., allowing input and output via choice of different modalities), effectiveness (*e.g*., using evidence-based intervention strategies), adoption (*e.g*., assistance in integrating the system into practice workflows and permitting customization of the website and feedback materials by practice teams), implementation (*e.g*., identifying and targeting actionable priority behavioral and psychosocial issues for patients and teams), and maintenance/sustainability (*e.g*., integration with current National Committee for Quality Assurance recommendations and clinical pathways of care). *Connection to Health *can work on a variety of input and output platforms, and assesses and provides feedback on multiple health behaviors and multiple chronic conditions frequently managed in adult primary care. As such, it should help to make patient-healthcare team encounters more informed and patient-centered. Formative research with clinicians indicated that the program addressed a number of practical concerns and they appreciated the flexibility and how the *Connection to Health *program could be customized to their office.

**Conclusions:**

This primary care practice tool based on an implementation science model has the potential to guide patients to more healthful behaviors and improved self-management of chronic conditions, while fostering effective and efficient communication between patients and their healthcare team. RE-AIM and similar models can help clinicians and media developers create practical products more likely to be widely adopted, feasible in busy medical practices, and able to produce public health impact.

## Background

The Institute of Medicine [1] outlined six criteria as the basis for preventive and chronic disease care: patient centered, effective, safe, timely, efficient, and equitable. One way of achieving these aims in primary care is by implementing the core criteria of the Patient-Centered Medical Home (PCMH), which has gained considerable traction as an important part of healthcare reform [2-4].

Achieving the aims of the PCMH, however, can be challenging due to the complexity and multiple competing demands on primary care. The PCMH model includes an emphasis on patient self-management support strategies that provide patients with the information, tools, and support they need to adopt healthy behaviors and take care of their health problems in their daily lives. However, primary care clinicians and staff often lack training in identifying and addressing health behavior and self-management support issues. Stange *et al*. [5] concluded that the average amount of time that primary care physicians can devote to prevention in a typical visit is one minute. Data documenting the routine adoption of these changes into primary care practice have been disappointing [6-17]; a large chasm remains between what is possible and what has been achieved [1]. To address this challenge, we describe an approach based on interactive behavior change technology (IBCT) as a vehicle for facilitating the adoption of PCMH strategies into primary care. The reach, effectiveness, adoption, implementation, maintenance/sustainability (RE-AIM) model [18,19] was used to develop the IBCT program to enhance its chances of successful adoption, implementation, and sustainability in primary care.

### Addressing primary care challenges

IBCT can provide efficient methods for achieving the goals of the PCMH. In a review of the literature, members of our team concluded that 'if constructed to draw on the strengths of primary care and to use patient-centered principles, IBCT can inform, leverage, and support patient-provider communication and enhance behavior change [20].' Integration of self-management support, a major component of the PCMH, into primary care practices can be facilitated through an easy-to-use, time-efficient IBCT system that addresses the most important, behavioral, and psychosocial challenges, especially if focused on the needs of patients with the most common chronic conditions.

The major goals of IBCT, which fit well with PCMH, are to: detect and then monitor patient needs for self-management support over time; prompt clinician/patient discussions to engage patients in behavior change; establish individualized priorities for identified problems; provide guidance and options for intervention at the point of care; and monitor success over time and prompt follow-ups [20,21]. However, to our knowledge no comprehensive system exists that includes prevention and multiple chronic disease monitoring and intervention that is based on practical, well-documented measures and directly tied to actionable resources and recommendations for clinicians and patients [22-32]. To date, IBCTs have not been widely adopted in real world primary care settings. We posit that one of the reasons for this may be that implementation science concerns and approaches like RE-AIM have not been integrated into the development and testing of the majority of IBCTs. In this article, we summarize key points of the RE-AIM implementation science model, and then describe how it was used to develop an IBCT for the PCMH [33,34].

The purposes of this article are to: describe the characteristics and design of the IBCT-based *Connection to Health *self-management support system to support the PCMH; illustrate the use of the RE-AIM model to guide development *of Connection to Health*; present qualitative results from a focus group discussion of *Connection to Health *with clinicians and staff members; and discuss practical implications and directions for future research and practice.

### RE-AIM planning and evaluation framework

RE-AIM was developed to help health planners and evaluators to attend to specific implementation factors essential for success in the real and complex world of healthcare and community settings [18,34]. It is an acronym that focuses attention on five key issues related to successful impact and can help design interventions that can: **r**each a broad and representative proportion of the target population; **e**ffectively lead to positive changes in patient self-management and quality of life that are robust across diverse groups; be **a**dopted across a broad and representative proportion of settings; lead to consistent **i**mplementation of strategies at a reasonable cost; and lead to **m**aintained self-management in patients and sustained delivery within primary care clinics [19,35,36].

RE-AIM can be a valuable planning tool for implementing self-management support and IBCT programs, especially considering the Institute of Medicine aims to provide efficient, patient-centered, equitable care and reduce health disparities. For example, a focus on the representativeness (*i.e*., reach) of those who engage with the technology and the robustness of the program's effect is critical. With this in mind, developers of an IBCT for self-management support should design features to ensure that appropriate audio and visual aids are in place to assist all patients, particularly low literacy, minority, less acculturated, older, poorer, or less educated patients who may feel overwhelmed with the healthcare system and confused by complex forms and procedures.

A focus on the RE-AIM factors of adoption, implementation, and sustainability of an IBCT self-management support system also addresses the larger issue of actionable information. With primary care already stretched beyond capacity to deal with care recommendations [5,37,38], adding additional assessment information will not solve the problem. Any additional information will need to be customized in ways that are compatible and integrated with practice flow, styles, priorities, and preferences to yield feasible, actionable outcomes. RE-AIM has previously been successfully applied to evaluate the impact of interactive technology approaches and clinic changes, providing an assessment of potential public health impact [20,39,40].

### Complexity

Many patients with chronic conditions experience major barriers to change related to ongoing co-morbid depression or disease-related distress, distinct conditions with different implications for care [41,42]. For example, depression is about twice as prevalent among patients with diabetes compared to community samples, and ongoing distress related to managing a demanding chronic disease like diabetes has an average prevalence rate of 18% to 35% [43]. Often, clinicians make recommendations for patients, only to see them not enacted because of feelings of hopelessness or being overwhelmed with the ongoing demands of chronic disease management. The delivery of actionable information must be tailored to the patient's capacity for change and the presence of emotional and distress-related barriers [41-43].

### Characteristics of the *Connection to Health *system

**Figure 1:**
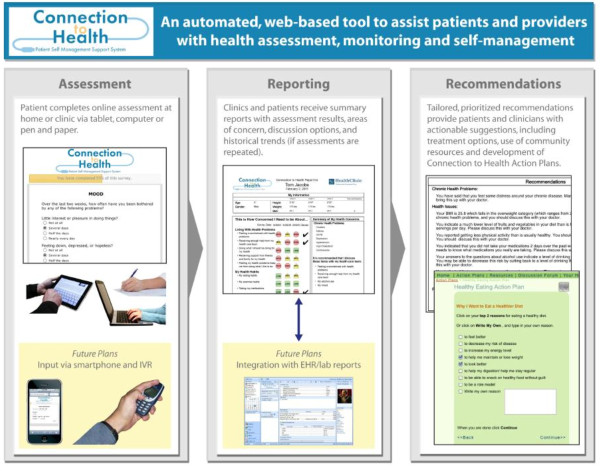
***Connection To Health *patient self-management support system**.

The *Connection to Health *Patient Self-Management System is designed to deliver an array of tools to assist patients and providers in the assessment, monitoring, and management of a variety of health behaviors, psychosocial concerns, and chronic disease problems. The automated, web-based system uses engaging graphics, multimedia, and educational design techniques, and database-driven responses to provide three primary modules to address patient interaction and self-management--ongoing patient assessment, delivering summary self-management support reports, and providing recommendations for patients and healthcare teams. The assessment module uses brief evidence-based screening scales to assess behaviors (including diet, tobacco use, risky drinking, physical activity, and medication adherence) and chronic conditions (including obesity, diabetes, coronary heart disease, hypertension, hyperlipidemia, asthma, stress, and depression). The reporting module offers summary reports to both clinicians and patients that include assessment results, areas of concern, discussion options, and patient trends over time. The recommendations module provides clinician and patient with patient-tailored and prioritized suggestions for action, including development of goals and action plans in a variety of health behavior and psychosocial domains. Clinics or practices that adopt the system can customize the *Connection to Health *website through an administrative portal to reflect their local identity and resources (Figure 1). The system is adaptable for integration with electronic health records (EHRs) so that the results can be shared easily across clinical team members, and patient self-management support status can be monitored over time.

### Welcome

1. The clinic uses the administrative portal to enter initial patient contact information into the *Connection to Health *database. The system then sends an e-mail or letter to the patient with an embedded link to the secure, Health Insurance Portability and Accountability Act (HIPAA)-compliant website. The patient clicks on the link and is presented with a multimedia (audio and/or video) welcome message designed to engage the user and encourage participation, including a message from the practice to indicate that the program is part of the care provided by their clinician.

### Assessment

Prior to each regularly scheduled chronic disease or preventive healthcare office visit, patients are prompted to complete a brief online assessment through the *Connection to Health *system. This assessment can be conducted through a patient portal to the website through a home computer, practice computer kiosk or pen tablet computer, or a paper-and-pencil application that can be scanned into the system.

### Reporting

**Figure 2:**
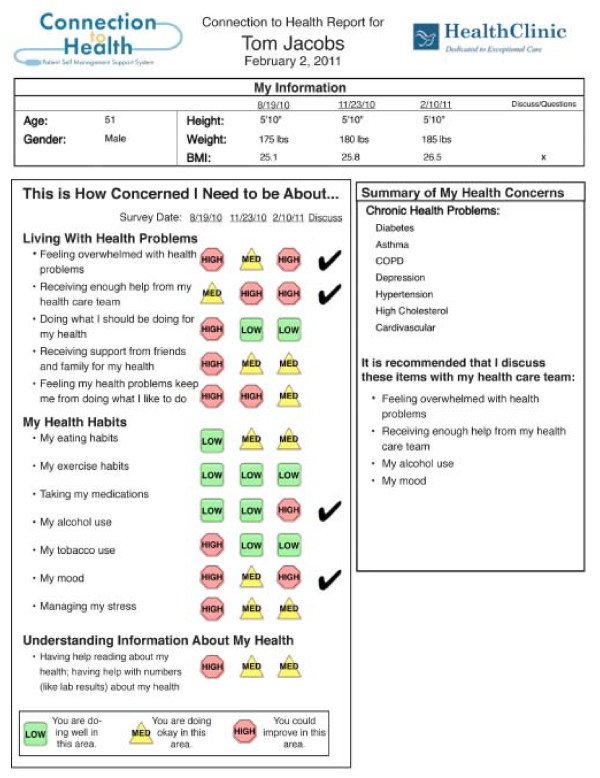
**Patient report**.

Once the patient has navigated through and completed the assessment module, the *Connection to Health *system uses validated algorithms to quickly score the assessments and display reports for both the patient and provider. The one-page patient report (example in Figure 2) can be viewed immediately through the patient portal or printed out hardcopy. It displays assessment results (including a history of recent assessments), areas of medical concern, and possible treatment options to discuss with the healthcare team. If the *Connection to Health *website is integrated with an EHR or laboratory reporting system, the patient report can also display selected, relevant laboratory results. The patient is encouraged to review the report, add her own notes or comments, and then have it sent or bring it to the next office visit or discussion with their clinician.

**Figure 3:**
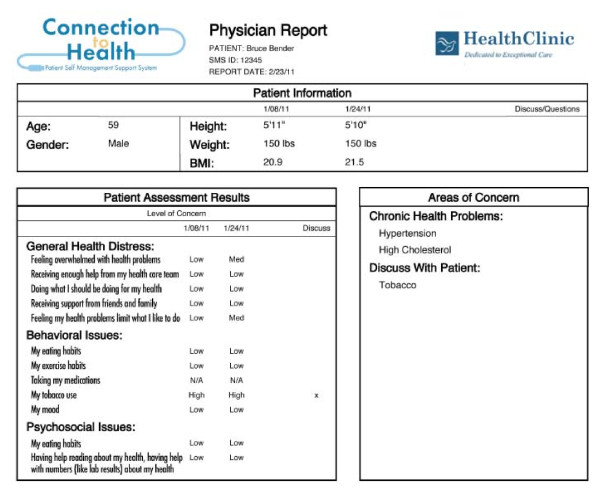
**Physician report**.

The physician report (Figure 3) contains much of the same information, but includes more details related to patient complexity, cardiovascular risk, health literacy and numeracy, and guideline concordant action recommendations. The goal of both reports is to provide an immediate, straightforward understanding of the patient's current health status; the self-management, psychosocial, and biologic areas of greatest patient concern; a prioritized list of items to discuss at the office visit; and an actionable set of self-management options and recommendations for flagged issues.

### Recommendations

Tailored recommendations for action, based on the results of the assessments, are included in the patient and provider reports. For example, if the patient scored low in physical activity and consumed many high fat foods and had a high low-density lipoprotein (LDL) reading, the recommendations might include tips for beginning a conversation about eating patterns and a *Connection to Health *action plan for healthful eating and physical activity. The primary care team can review the patient and physician reports prior to the office visit, providing the primary care physician (PCP) with a concise set of assessment results and treatment options and tips for guiding the discussion with the patient.

The *Connection to Health *action plan module, available through the patient portal, provides a strategy for patient self-management that can be selected for use with patients who would respond to an interactive web-based action planning program and/or in situations where the practice does not have the time or appropriate staffing to complete the action planning process. This area of the website is derived from our series of successful interventions based upon problem-solving theory [44,45]. This section offers engaging multimedia modules that guide the user through an action planning process for selected key health behaviors, including diet, exercise, medication adherence, smoking cessation, alcohol use, and depression/distress. These interactive modules facilitate patient selection of goals in any of these areas, and identification of benefits, barriers to success, and strategies for overcoming these barriers. The *Connection to Health *action plan module stores patient action plans and provides ongoing access to the plans by the healthcare team and the patient for self-monitoring and follow-up. Alternatively, the healthcare team may decide to provide intervention resources in person in the clinic or to refer the patient to a community resource (*e.g*., YMCA programs, voluntary associations, telephone help lines, or quit smoking cessation resources).

### Follow-up

The *Connection to Health *System provides ongoing monitoring and prompts follow-up by both the patient and the practitioner. The self-monitoring component allows the patient to track their progress over time. Shortly before the patient is scheduled for another visit to the clinic or practice, he or she can be prompted to complete another set of brief assessments in advance of that visit and to review their history and progress.

### Current *Connection to Health *measures

In choosing areas for screening and more in-depth assessment, we selected measures that address prevalent conditions or problems that have large public health impact, considered participant burden, and lead to actionable outcomes. Congruent with the recent policy recommendation from the Society of Behavioral Medicine http://www.sbm.org/policy/patient-reported_measures.pdf, we emphasized brief scales that were reliable, sensitive to change, appropriate for repeated administration, and age appropriate [46]. As can be seen in Figures 1 and 2, *Connection to Health *currently includes assessments for depression, disease-related distress, medication adherence, smoking, physical activity, risky drinking, eating patterns, current stressors, and health literacy and numeracy. In addition, questions related to the patient's chronic diseases assess aspects of their management of those conditions. Additional file 1, Appendix 1 provides a brief summary of each instrument included in the *Connection to Health *assessment package.

### Use of RE-AIM for *Connection to Health *development

We used the RE-AIM model [19,33,35] in developing the *Connection to Health *tool, by applying it to the goals of the PCMH. Table 1 summarizes how we addressed each of the RE-AIM elements.

**Table 1:** Use of RE-AIM to develop *Connection to Health *PCMH tool

RE-AIM dimension	Ways dimension was used to enhance impact
Reach	Multiple input modalities; patient choice, panel report so can target those not participating

Effectiveness	Practical, validated, actionable measures, evidence-based action suggestions. Patient choice to enhance autonomy. Expert system tailoring algorithms. Use of 5 A's, goal setting, and action planning problem solving.

Adoption	Specifically designed to the support PCMH. Multiple options for customization of input, output, content, and recommended options. Panel reports for population management. Addresses HEDIS-related issues often missed.

Implementation	Focus on efficiency, prompts to patient and healthcare team, done prior to visit, self-monitoring elements, engaging interface. Options for high and low eHealth literacy.

Maintenance	Setting Level: Feedback on HEDIS and PCMH criteria. Should enhance satisfaction and make visits more efficient and productive. Patient Level: Should enhance continuity, patient-provider communication and satisfaction.

### Reach

*Connection to Health *is designed to have high reach through several design features, including multiple modalities for data input and output. Patients can be provided with their choice of entry modality, and systems can be created to ensure that the entire patient panel of the practice is screened. Future iterations of *Connection to Health *will be designed with the capability to also accept data from automated telephone calls, cell phone data entry, a personal health record or EHR, and future data entry modalities.

### Effectiveness

Effectiveness is enhanced in multiple ways: use of practical, validated scales and measures [46-49]; links to evidence-based electronic and community resources; and patient choice at multiple steps in the process [50]. Patient choice has been shown to be related to enhanced perceptions of autonomy support and improved outcomes [50]. We also use expert system tailoring [51,52] to select tailored intervention strategies based upon key behavioral and psychosocial factors. The system can easily be enhanced or modified overtime by adding in additional relevant local self-management support resources or other evidence-based links or information.

### Adoption

*Connection to Health *offers practices numerous incentives for adoption, providing techniques and options to assist practices in goals related to enhancing patient-centeredness, a primary goal of PCMH. Assessments can be completed before or after office visits, thus not taking any office time or interfering with patient flow. It addresses psychosocial issues such as distress and depression/anxiety, includes an efficient method for helping patients to prioritize their goals and questions, helps patients attend office visits well-prepared and engaged, and by doing so, saves practices time and increases efficiency. The use of *Connection to Health *also could assist the practice in meeting the standards for recognition as a PCMH and improve quality measures.

### Implementation

Being automated, *Connection to Health *ensures consistent delivery, accurate scoring, and immediate reporting of results. The administrative report feature enhances implementation by providing regular patient and panel-level reports at intervals specified by the practice and documents improvement over time.

### Maintenance

Helping practices achieve, and be reimbursed for, higher performance on PCMH and quality measures should enhance maintenance. Maintenance at the patient level is enhanced by increased goal accomplishment, regular follow-up and feedback, and self-monitoring of individually targeted behaviors [53-55].

### Initial provider reactions to *Connection to Health*

The initial version of the *Connection to Health *Patient self-management support was presented to a focus group of clinicians and staff from 10 family medicine practices working on implementation of the PCMH model. Field notes were taken by the two facilitators, and the participants also provided written comments using a structured format.

Feedback was very positive, providing important input regarding the assessment, the practice reports, and the potential implementation of the system in their practices. Comments highlighted the following issues:

1. Clinicians particularly liked that this system is designed to assist in focusing discussions of self-management issues between clinicians and patients and not to be a stand-alone system. They indicated that if the system was automated outside the practice, they believed that it would not be successful due to lack of reinforcement by the primary care clinicians.

2. Clinicians could be resistant because the system might cause them to feel separated from their patients. However, if the system is well-integrated within the practice, it will need to be done is a manner that minimizes the time commitment.

3. The flexibility and ability to customize the *Connection to Health *to fit needs, patient flows, and preferences of local clinics should aid adoption. Practices will have varying personnel and workflow that will necessitate different strategies for implementing the *Connection to Health *system at different points in patient flow and using different modalities in different practices.

4. Clinicians that have an EHR would like a seamless interface of the *Connection to Health *system with their system, while recognizing that will be a challenge.

5. Clinicians wanted to be shown how *Connection to Health *can be time-efficient

## Discussion

Most self-management support programs address a single disease or single behavior, and few are designed for primary care practices [51,56]. In contrast, *Connection to Health *has broad applicability across diseases, prevention, multiple behaviors, and varied primary care settings for a wide range of adult patients. It can be accessed through several modalities and is appropriate for patients with diverse socioeconomic and educational backgrounds. It is designed to be integrated into primary care, creating efficiency while prompting informed provider-patient communication. *Connection to Health *should support the PCMH, create more informed and efficient office visits, and prompt and promote critical but often not completed follow-up support.

The primary purposes of this paper were to describe the *Connection to Health *system and how the RE-AIM framework was used proactively to develop it. Although controlled and comparative effectiveness studies are needed to determine the ultimate impact of the *Connection to Health*, use of implementation science models such as RE-AIM or other dissemination frameworks at the design stage [57,58] should greatly facilitate greater uptake, implementation success, and long-term results. The *Connection to Health *is intentionally a work in progress, with iterative improvements to be made in the selection of measurement items and domains, patient and provider interfaces, and data input and output modalities.

*Connection to Health *is to our knowledge the only tool for addressing a wide variety of prevalent behavioral, psychosocial, and disease management problems managed in primary care. Time-efficient tools such as *Connection to Health *can help both patients and healthcare team members come to interactions more informed and prepared. This, in turn, should improve both outcomes and satisfaction [21,25,26,59]. Finally, the panel management features of the *Connection to Health *should facilitate continuity of care and consistent follow-up, which is the element of care recommendations least often accomplished [60,61].

Potential limitations include that the *Connection to Health *system is likely only appropriate for adult primary care patients, and not for children and adolescents (different measures would be needed). Currently, it is available only in English. Although computer administered, including automated skip patterns and individualized tailoring, it does not employ item response theory or formal computer-assisted testing procedures http://www.nihpromis.org/default.aspx. It is also possible that with repeated use over time that patients would begin to find the assessment process burdensome, and a *Connection to Health *quick-scan form may need to be developed for prevalent, well-defined subgroups of patients (*e.g*., overweight diabetes patients). The degree to which active follow-up with a patient within the PCMH model could overcome this limitation is an area ripe for investigation. Finally, although we found the RE-AIM model useful for planning and developing *Connection to Health*, other implementation science models could also have been used and RE-AIM does not explicitly address some issues such as stakeholder engagement. Readers interested in applying RE-AIM for program development and planning purposes should find the resources listed in Table 2 helpful for gaining a more complete understanding of the model and its implications.

**Table 2:** Key RE-AIM Publications by Implementation Topic

Issue or Topic	RE-AIM Resource
Original Source	Glasgow RE, Vogt TM, Boles SM. Evaluating the public health impact of health promotion interventions: The RE-AIM framework. Am J Public Health 1999;89:1322-7 [18].
Use in Planning	Klesges LM, Estabrooks PA, Glasgow RE, Dzewaltowski D. Beginning with the application in mind: Designing and planning health behavior change interventions to enhance dissemination. Ann Behav Med 2005;29((Suppl)):66S-75S [58].
Prevention Application	Glasgow RE, Vogt TM, Boles SM. Evaluating the public health impact of health promotion interventions: The RE-AIM framework. Am J Public Health 1999;89:1322-7 [18].
Treatment Application	Glasgow RE, Nutting PA, King DK, Nelson CC, Cutter G, Gaglio B, Rahm AK, Whitesides H. Randomized effectiveness trial of a computer-assisted intervention to improve diabetes care. Diabetes Care 2005 January;28(1):33-9 [62].
RE-AIM Measures	Glasgow RE, Nelson CC, Strycker LA, King DK. Using RE-AIM metrics to evaluate diabetes self-management support interventions. Am J Prev Med 2006;30(1):67-73 [63].
Primary Care Application	Glasgow RE. RE-AIMing research for application: Ways to improve evidence for family practice. Journal of the American Board of Family Practice 2006;19(1):11-9 [36].
Health Technology Applications	Glasgow RE, McKay HG, Piette JD, Reynolds KD. The RE-AIM framework for evaluating interventions: What can it tell us about approaches to chronic illness management? Patient Educ Couns 2001;44:119-27 [35].
	Glasgow RE, Bull SS, Piette JD, Steiner J. Interactive behavior change technology: A partial solution to the competing demands of primary care. Am J Prev Med 2004;27(25):80-7 [20].
Policy Application	Jilcott S, Ammerman C, Sommers J, Glasgow RE. Applying the RE-AIM framework to assess the public health impact of policy change. Ann Behav Med 2007;34(2):105-14 [64].
Community Application	Estabrooks PA, Bradshaw M, Dzewaltowski DA, Smith-Ray RL. Determining the impact of Walk Kansas: applying a team-building approach to community physical activity promotion. Ann Behav Med 2008 August;36(1):1-12 [65].
Environmental Change	King DK, Glasgow RE, Leeman-Castillo B. RE-AIMing RE-AIM: Using the model to plan, implement, evaluate, and report the impact of environmental change approaches to enhance population health. Am J Public Health. 2010: 2076-2084 [66].
Tools, Quizzes, Training, etc.	http://www.re-aim.org RE-AIM online training at http://www.trt.org
Overall Summary Resources	Gaglio B, Glasgow RE. Evaluation Approaches for Dissemination and Implementation Research. In: Brownson R, editor. Dissemination and Implementation Research. In press ed. In press: 2011 [34]. http://www.re-aim.org

Future research should evaluate and document the actual use, time efficiency, multifaceted impact, reach or percent and characteristics of patients who can be assessed with it, and its actual implementation in primary care, using RE-AIM [34] or other implementation science models. In particular, comparative effectiveness research studies are indicated to determine, for example, if the *Connection to Health *is more cost-effective than alternatives, such as simple paper and pencil assessments followed by more traditional face-to-face interventions. Practical implications are that implementation science models, such as RE-AIM, should be employed throughout the design process to maximize impact.

## Competing interests

All authors declare no competing interests. The *Connection to Health *Patient Self-management Support System is intended to provide a platform to improve prevention and self-management quality within healthcare or other health promoting entities. Our intent is to make any programs developed from our research in this area freely accessible to health delivery systems to allow for broad dissemination and use. Any proceeds secured from *Connection to Health *self-management support will be used for continued research and development and will not be used to generate individual income.

## Authors' contributions

All authors have made substantial contributions to conception and design, or acquisition of data, or analysis and interpretation of data, have been involved in drafting the manuscript or revising it critically for important intellectual content, and have given final approval of the version to be published.

## Funding

The Colorado Health Foundation and Robert Wood Johnson Foundation provided funding that supported a portion of the planning and development of the *Connection to Health *Patient Self-management System.

## Disclosure

Dr. Glasgow is now employed at the National Cancer Institute (NCI). This work was completed before he transitioned to the NCI and the opinions expressed do not necessarily reflect those of the NCI.

## Supplementary Material

Additional file 1 **Appendix 1. Measures: Scales chosen, rationale for selection, actionable results**. Click here for file

## References

[B1] Institute of Medicine, Committee on Quality Health Care in America Crossing the quality chasm: A new health system for the 21st century 2003 Washington, DC: National Academies Press

[B2] Grundy P Hagan KR Hansen JC Grumbach K The multi-stakeholder movement for primary care renewal and reform Health Aff (Millwood) 2010 29 5 791 8 10.1377/hlthaff.2010.0084 20439863

[B3] Bodenheimer t Pham HH Primary care: current problems and proposed solutions Health Aff (Millwood) 2010 29 5 799 805 10.1377/hlthaff.2010.0026 20439864

[B4] Reid RJ Coleman K Johnson EA Fishman PA Hsu C Soman MP Trescott CE Erikson M Larson EB The group health medical home at year two: cost savings, higher patient satisfaction, and less burnout for providers Health Aff (Millwood) 2010 29 5 835 43 10.1377/hlthaff.2010.0158 20439869

[B5] Stange KC Woolf SH Gjeltema K One minute for prevention: The power of leveraging to fulfill the promise of health behavior counseling Am J Prev Med 2002 22 320 3 10.1016/S0749-3797(02)00413-0 11988386

[B6] Baron RJ Desnouee E Practice profile. The struggle to support patients' efforts to change their unhealthy behavior Health Aff (Millwood) 2010 29 5 953 5 10.1377/hlthaff.2010.0053 20439886

[B7] Beckles GL Engelgau MM Narayan KM Herman WH Aubert RE Williamson DF Population-based assessment of the level of care among adults with diabetes in the U.S Diabetes Care 1998 21 9 1432 8 10.2337/diacare.21.9.1432 9727887

[B8] Saaddine JB Engelgau MM Beckles GL Gregg EW Thompson TJ Narayan KM A diabetes report card for the United States: Quality of care in the 1990s Ann Intern Med 2002 136 8 565 74 11955024 10.7326/0003-4819-136-8-200204160-00005

[B9] Greenfield S Kaplan SH Kahn R Ninomiya J Griffith JL Profiling care provided by different groups of physicians: effects of patient case-mix (bias) and physician-level clustering on quality assessment results Ann Intern Med 2002 136 2 111 21 11790062 10.7326/0003-4819-136-2-200201150-00008

[B10] Porterfield DS Kinsinger L Quality of care for uninsured patients with diabetes in a rural area Diabetes Care 2002 25 2 319 23 10.2337/diacare.25.2.319 11815503

[B11] Srinivasan M Przybylski M Swigonski N The Oregon Health Plan: predictors of office-based diabetic quality of care Diabetes Care 2001 24 2 262 7 10.2337/diacare.24.2.262 11213876

[B12] Acton KJ Shields R Rith-Najarian S Tolbert B Kelly J Moore K Valdez L Skipper B Gohdes D Applying the diabetes quality improvement project indicators in the Indian Health Service primary care setting Diabetes Care 2001 24 1 22 6 10.2337/diacare.24.1.22 11194234

[B13] Chin MH Zhang JX Merrell K Diabetes in the African-American Medicare population Diabetes Care 1998 21 7 1090 5 10.2337/diacare.21.7.1090 9653601

[B14] Davidson MB Diabetes care in health maintenance organisation and fee-for-service settings Disease Management and Health Outcomes 1997 2 189 97 10.2165/00115677-199702040-00003

[B15] Davidson MB The case for 'outsourcing' diabetes care Diabetes Care 2003 26 1608 12 10.2337/diacare.26.5.1608 12716826

[B16] Suwattee P Lynch JC Pendergrass ML Quality of care for diabetic patients in a large urban public hospital Diabetes Care 2003 26 3 563 8 10.2337/diacare.26.3.563 12610002

[B17] Bell RA Camacho F Goonan K Duren-Winfield V Anderson RT Konen JC Goff DC Jr Quality of diabetes care among low-income patients in North Carolina Am J Prev Med 2001 21 2 124 31 10.1016/S0749-3797(01)00328-2 11457632

[B18] Glasgow RE Vogt TM Boles SM Evaluating the public health impact of health promotion interventions: The RE-AIM framework Am J Public Health 1999 89 1322 7 PMID 10474547 10.2105/AJPH.89.9.1322 10474547 PMC1508772

[B19] Gaglio B Glasgow RE Brownson R Evaluation Approaches for Dissemination and Implementation Research Dissemination and Implementation Research 2010 in press

[B20] Glasgow RE Bull SS Piette JD Steiner J Interactive behavior change technology: A partial solution to the competing demands of primary care Am J Prev Med 2004 27 25 80 7 15275676 10.1016/j.amepre.2004.04.026

[B21] Glasgow RE Interactive media for diabetes self-management: Issues in maximizing public health impact Medical Decision Making 2010 30 6 745 58 10.1177/0272989X10385845 21183760

[B22] Glasgow RE Hiss RG Anderson RM Friedman NM Hayward RA Marrero DG Taylor CB Vinicor F Report of Health Care Delivery Work Group: Behavioral research related to the establishment of a chronic disease model for diabetes care Diabetes Care 2001 24 124 30 10.2337/diacare.24.1.124 11194217

[B23] Hiss RG Barriers to care in non-insulin-dependent diabetes mellitus, The Michigan experience Ann Intern Med 1996 124 1 Part 2 146 8 8554207 10.7326/0003-4819-124-1_part_2-199601011-00012

[B24] Wagner EH Austin B Von Korff M Improving outcomes in chronic illness Manag Care Q 1996 4 2 12 25 10157259

[B25] Wagner EH Davis C Schaefer J Von Korff M Austin B A survey of leading chronic disease management programs: Are they consistent with the literature? Manag Care Q 1999 7 3 56 66 10620960

[B26] Wagner EH Austin BT Von Korff M Organizing care for patients with chronic illness Milbank Quarterly 1996 74 511 44 10.2307/3350391 8941260

[B27] Cabana M Rand CS Powe NR Wu AW Wilson MH Abboud PA Rubin HR Why don't physicians follow clinical practice guidelines?: A framework for improvement JAMA 1999 282 1458 65 10.1001/jama.282.15.1458 10535437

[B28] Norris SL Engelgau MM Narayan KM Effectiveness of self-management training in type 2 diabetes: Systematic review of randomized controlled trials Diabetes Care 2001 24 3 561 87 10.2337/diacare.24.3.561 11289485

[B29] Polonsky WH Earles J Smith S Pease DJ Macmillan M Christensen R Taylor T Dickert J Jackson RA Integrating medical management with diabetes self-management training: a randomized control trial of the Diabetes Outpatient Intensive Treatment program Diabetes Care 2003 26 11 3048 53 10.2337/diacare.26.11.3048 14578238

[B30] Norris SL Nichols PJ Caspersen CJ Glasgow RE Engelgau MM Jack L Snyder SR Carande-Kulis VG Isham G Garfield S Briss P McCulloch D Increasing diabetes self-management education in community settings. A systematic review Am J Prev Med 2002 22 4 Supp 39 66 11985934 10.1016/s0749-3797(02)00424-5

[B31] Clark NM Becker MH Lorig K Rakowski W Anderson L Self-management of chronic disease by older adults: A review and questions for research J Aging Health 1991 3 1 3 27 10.1177/089826439100300101

[B32] Lorig KR Sobel DS Stewart AL Brown BW Bandura A Ritter P Gonzalez VM Laurent DD Holman HR Evidence suggesting that a chronic disease self-management program can improve health status while reducing hospitalization: A randomized trial Med Care 1999 37 1 5 14 10.1097/00005650-199901000-00003 10413387

[B33] Glasgow RE Linnan LA Glanz K, Rimer BK, Viswanath K Evaluation of theory-based interventions Health Behavior and Health Education: Theory, Research, and Practice 2008 4 San Francisco, CA: Jossey-Bass 487 508

[B34] Gaglio B Glasgow RE Brownson R Evaluation Approaches for Dissemination and Implementation Research Dissemination and Implementation Research 2011 in press

[B35] Glasgow RE McKay HG Piette JD Reynolds KD The RE-AIM framework for evaluating interventions: What can it tell us about approaches to chronic illness management? Patient Educ Couns 2001 44 119 27 PMID 11479052 10.1016/S0738-3991(00)00186-5 11479052

[B36] Glasgow RE RE-AIMing research for application: Ways to improve evidence for family practice Journal of the American Board of Family Practice 2006 19 1 11 9 PMID: 16492000 10.3122/jabfm.19.1.11 16492000

[B37] Yarnell KS Pollack KI Ostbye T Krause KM Michener JL Primary care: Is there enough time for prevention? Am J Public Health 2003 93 4 635 41 10.2105/AJPH.93.4.635 12660210 PMC1447803

[B38] Ostbye T Yarnall KS Krause KM Pollak KI Gradison M Michener JL Is there time for management of patients with chronic diseases in primary care? Ann Fam Med 2005 3 3 209 14 10.1370/afm.310 15928223 PMC1466884

[B39] Glasgow RE Toobert DJ Hampson SE Strycker LA Implementing, generalization and long-term results of the 'choosing well' diabetes self-management intervention Patient Educ Couns 2001 48 2 115 22 10.1016/s0738-3991(02)00025-3 12401414

[B40] Glasgow RE Nutting PA King DK Nelson CC Cutter G Gaglio B Kulchak Rahm A Whitesides H Amthauer H A practical randomized trial to improve diabetes care J Gen Intern Med 2004 19 12 1167 74 PMID 15610326 10.1111/j.1525-1497.2004.30425.x 15610326 PMC1492587

[B41] Fisher L Skaff MM Mulllan JT Arean P Mohr D Masharani U Glasgow R Laurencin G Clinical depression versus distress among patients with type 2 diabetes: Not just a question of semantics Diabetes Care 2007 30 3 542 8 10.2337/dc06-1614 17327318

[B42] Fisher L Skaff MM Mullan JT Arean P Glasgow R Masharani U A longitudinal study of affective and anxiety disorders, depressive affect and diabetes distress in adults with type 2 diabetes Diabetic Med 2008 25 1096 101 10.1111/j.1464-5491.2008.02533.x 19183314 PMC2635496

[B43] Fisher L Mullan JT Arean P Glasgow RE Hessler D Masharani U Diabetes distress and not clinical depression or depressive affect is associated with glycemic control in both corss-sectional and longitudinal analyses Diabetes Care 2010 33 23 8 10.2337/dc09-1238 19837786 PMC2797978

[B44] Glasgow RE Kurz D King DK Dickman JM Faber AJ Halterman E Wooley T Toobert DJ Strycker LA Estabrooks PA Osuna D Ritzwoller D Outcomes of a minimal versus moderate support versions of an internet-based diabetes self-management support program J Gen Int Med 2010 25 12 1315 22 10.1007/s11606-010-1480-0 PMC2988142 20714820

[B45] Toobert DJ Strycker LA Barrera M Jr Osuna D King DK Glasgow RE Outcomes from a Multiple-Risk-Factor Diabetes Self-Management Trial for Latinas: ¡Viva Bien! Trans Behav Med 2011 41 3 310 23 10.1007/s12160-010-9256-7 PMC3108326 21213091

[B46] Glasgow RE Ory MG Klesges LM Cifuentes M Fernald DH Green LA Practical and relevant self-report measures of patient health behaviors for primary care research Annals of Family Medicine 2005 3 73 81 10.1370/afm.261 15671195 PMC1466792

[B47] Paxton A Strycker LA Toobert DJ Ammerman AS Glasgow RE Starting the Conversation: Performance of a brief dietary assessment and intervention tool for health professionals Am J Prev Med 2010 40 1 67 71 10.1016/j.amepre.2010.10.009 21146770

[B48] Kroenke K Spitzer RL Williams JB The PHQ-9: Validity of a brief depression severity measure J Gen Intern Med 2001 16 9 606 13 10.1046/j.1525-1497.2001.016009606.x 11556941 PMC1495268

[B49] Kroenke K Spitzer RL The PHQ-9: A new depression diagnostic and severity measure Psychiatr Ann 2002 32 9 1 7

[B50] Williams GC Lynch M Glasgow RE Computer-assisted intervention improve patient-centered diabetes care by increasing autonomy support and perceived competence Health Psychol 2007 26 6 728 34 18020845 10.1037/0278-6133.26.6.728

[B51] Strecher V Internet methods for delivering behavioral and health-related interventions (eHealth) Annu Rev Clin Psychol 2007 3 53 76 10.1146/annurev.clinpsy.3.022806.091428 17716048

[B52] Kreuter MW Wray RJ Tailored and targeted health communication: strategies for enhancing information relevance Am J Health Behav 2003 27 Suppl 3 S227 S232 14672383 10.5993/ajhb.27.1.s3.6

[B53] Wing RR Hill JO Successful weight loss maintenance Annu Rev Nutr 2001 21 323 41 10.1146/annurev.nutr.21.1.323 11375440

[B54] Phelan S Liu T Gorin A Lowe M Hogan J Fava J Wing RR What distinguishes weight-loss maintainers from the treatment-seeking obese? Analysis of environmental, behavioral, and psychosocial variables in diverse populations Ann Behav Med 2009 38 2 94 104 10.1007/s12160-009-9135-2 19847584 PMC4861315

[B55] Wing RR Hill JO Successful weight loss maintenance Ann Rev Nutr 2001 21 323 41 10.1146/annurev.nutr.21.1.323 11375440

[B56] Bennett GG Glasgow RE The delivery of public health interventions via the Internet: Actualizing their potential Annu Rev Public Health 2009 30 273 92 10.1146/annurev.publhealth.031308.100235 19296777

[B57] Kerner J Rimer B Emmons K Introduction to the special section on dissemination: Dissemination research and research dissemination: How can we close the gap? Health Psychol 2005 24 5 443 6 16162037 10.1037/0278-6133.24.5.443

[B58] Klesges LM Estabrooks PA Glasgow RE Dzewaltowski D Beginning with the application in mind: Designing and planning health behavior change interventions to enhance dissemination Ann Behav Med 2005 29 Suppl 66S 75S PMID 15921491 10.1207/s15324796abm2902s_10 15921491

[B59] Wagner EH Austin BT Davis C Hindmarsh M Schaefer J Improving chronic illness care: Translating evidence into action Health Aff 2001 20 64 78 10.1377/hlthaff.20.6.64 11816692

[B60] Glasgow RE Eakin EG Fisher EB Bacak SJ Brownson RC Physician advice and support for physical activity: Results from a national survey Am J Prev Med 2001 21 3 189 96 10.1016/S0749-3797(01)00350-6 11567839

[B61] Glasgow RE Wagner E Schaefer J Mahoney L Reid R Greene S Development and validation of the Patient Assessment of Chronic Illness Care (PACIC) Med Care 2005 43 5 436 44 10.1097/01.mlr.0000160375.47920.8c 15838407

[B62] Glasgow RE Nutting PA King DK Nelson CC Cutter G Gaglio B Rahm AK Whitesides H Randomized effectiveness trial of a computer-assisted intervention to improve diabetes care Diabetes Care 2005 28 1 33 9 10.2337/diacare.28.1.33 15616230

[B63] Glasgow RE Nelson CC Strycker LA King DK Using RE-AIM metrics to evaluate diabetes self-management support interventions Am J Prev Med 2006 30 1 67 73 PMID 16414426 10.1016/j.amepre.2005.08.037 16414426

[B64] Jilcott S Ammerman C Sommers J Glasgow RE Applying the RE-AIM framework to assess the public health impact of policy change Ann Behav Med 2007 34 2 105 14 10.1007/BF02872666 17927550

[B65] Estabrooks PA Bradshaw M Dzewaltowski DA Smith-Ray RL Determining the impact of Walk Kansas: applying a team-building approach to community physical activity promotion Ann Behav Med 2008 36 1 1 12 10.1007/s12160-008-9040-0 18607666

[B66] King DK Glasgow RE Leeman-Castillo B RE-AIMing RE-AIM: Using the model to plan, implement, evaluate, and report the impact of environmental change approaches to enhance population health Am J Public Health 2010 100 2076 2084 10.2105/AJPH.2009.190959 20864705 PMC2951937

