# Supplementary material for: Use of RE-AIM to develop a multi-media facilitation tool for the patient-centered medical home
Source: Implement Sci. 2011 Oct 21;6:118. doi: 10.1186/1748-5908-6-118 (PMC3229439; doi:10.1186/1748-5908-6-118)
Supplement: Additional file 1 — Appendix 1. Measures: Scales chosen, rationale for selection, actionable results. [file 1748-5908-6-118-S1.DOC]

| **Appendix 1**  Measures: Scales chosen, rationale for selection, actionable results | | | | |
| --- | --- | --- | --- | --- |
| Health Behavior/Psychosocial Area | | Current Measure | Why Instrument Chosen | Actionable Results (for Patient Meeting Criteria for Potential Problem) |
| Patient Characteristics | |  |  |  |
|  | Race/Ethnicity | Based on U.S. Department Health and Human Services (HHS) definitions. | To ensure ultimate accessibility of program in diverse populations. | NA |
|  | Education (years in school) | Self-report | To ensure ultimate accessibility of program for all education levels. | NA |
|  | Age | Date of Birth | To ensure ultimate accessibility of program for adults of all ages. | NA |
|  | Gender | Self-Report | To ensure ultimate accessibility of program for both genders. | NA |
|  | Height , Weight | Electronic medical record, and patient self-report. Compute body mass index. | For demonstration of improvement in patient health outcomes. | BMI in the overweight or obese category indicates:   1. Discuss with PCP 2. Set diet and physical activity goals. |
| Patient Identification |  |  |  |  |
|  | Practice (from list of involved practices)  Patient name | NA | For program tracking | NA |
| Chronic Disease | Presence or absence of diabetes, pre-diabetes, asthma, Chronic lung disease (COPD or emphysema),  Depression, High blood pressure (hypertension), High cholesterol or high lipids, Cardiovascular disease (including angina, heart attack, blocked heart artery), Arthritis, Other chronic illness. | CHRONIC DISEASES  Have you been told by a doctor that you have any of the following conditions? (followed by list of chronic diseases addressed in the *CTH* program). | Self-report | The patient reports having the following chronic health problems:   1. List in physician printout. 2. Ask disease specific follow up questions. |
| General health distress |  | Four items from the Modified Diabetes Distress Scale[41-43]  (revised for chronic illness rather than specific to diabetes). | Assessment of patient concerns about disease management, support, emotional burden, and access to care. | If patient has disease specific distress:   1. Encourage brief discussion with PCP. 2. Simplify diabetes regimen. 3. Refer to community resources. |
| Eating patterns |  |  |  |  |
|  | High-fat foods | Two items modified from Summary of Diabetes Self-Care Activities Questionnaire [62] with typical portion size assessment. | Chosen for brevity, and wide-spread use in diabetes populations; generalizable to other chronic conditions. | 1. Recommend substantial reduction of fat intake, and/or portion control of high-fat food items. 2. Set fat reduction goal. 3. Recommend nutritional education (office, referral,   Connection to Health website, or community resource). |
|  | Fruits and vegetables | Two items Modified from Summary of Diabetes Self-Care Activities Questionnaire[62] with typical portion size assessment. | Chosen for brevity, and wide-spread use in diabetes populations. | 1. Recommend increase to five or more servings of fruits and vegetables 2. Set fruit and vegetable goals. 3. Recommend nutritional education (office, referral, Connection to Health website, or community resource). |
|  | Soda | One item from Starting the Conversation[63] | Brief, simple, clear | 1. Recommend setting goal to decrease to 12 or fewer ounces of soda intake per day 2. Recommend discussion with PCP or nutrition educator. |
|  | Salt | Two items from NHANES 2005-06 [64] . | The salt questions include use of salt at the table, use of salt in food preparation, intake of canned foods (not low sodium) and intake of packaged foods. | 1. Discuss with PCP (increased risk for health problems). 2. Set goal to reduce salt intake to <2300mg per day ; <1,500 mg per day if >.51 yrs, have HTN, DM or CKD. 3. Refer to nutrition educator. |
| Physical activity |  | The Modified International Physical Activity Questionnaire (IPAQ)[65] | Chosen because the IPAQ focuses on minutes of "moderate" activity rather than focusing on vigorous activity. | 1. PCP recommends patient increase moderate physical activity to 150 minutes per week. 2. Recommend further action planning. 3. Refer to community resources. |
| Medication adherence |  | The first question queries whether or not patients skip taking prescribed medications. This is followed by 5 items the respondent can endorse indicating reasons for skipping medications (cost, forgetting, side effects, not helping, unsure of reason they are taking medications) [66-68]. | The basic premise of asking patients to place a relative estimate of their degree of adherence exists in a variety of forms. | 1. Suggest patient discuss with PCP. 2. These “barriers” predict medication-taking, and PCP can use them as "talking points." |
| Alcohol |  | Two alcohol-related items from the set known as the “moderate drinking guidelines” [69;70]. | These two items were chosen based on epidemiologic research defined levels of alcohol consumption that are generally safe for most people. They also are recommended by the NIAAA and the SBIRT program (Screening and Brief Intervention, Referral to Treatment) for screening purposes and were the screening items recommended as part of the Colorado Clinical Guideline Collaboratives Alcohol and Substance Abuse Guideline. | 1. PCP recommends abstinence if patient is pregnant. 2. Recommend setting goals within moderate drinking guidelines. 3. Remind PCP of risk associated with heavy drinking. |
| Tobacco |  | One item from the BRFSS [71] assessing whether or not the patient uses tobacco, and if so, 3 follow-up items [72] to determine the amount and type of tobacco used (http://hmcrc.srph.tamhsc.edu/ Measures/TobaccoSNT.pdf). | Recommended as a practical and reliable self report instrument by Prescription for Health, a program of the Robert Wood Johnson Foundation and the Agency for Healthcare Research and Quality [46]. | 1. Short discussion with patient suggesting smoking cessation. 2. Suggest community resource (e.g., Quitline); screen for COPD. |
| Depression |  | 8 items from the PHQ-8 [73]. | Has been shown to have excellent characteristics for assessing depressive and anxiety  disorders. | 1. Recommend patient discuss with PCP.   Referral to community resource. |
| Chronic/acute stressors |  | One item culled from the life event literature, and the chronic/acute stress literature. | This item queries stress due to a major change or event or from a stressful situation (for example, family, work, or financial problems). | 1. Recommend discussion with PCP. 2. Evaluate in context of other issues identified in screener. |
| Literacy |  | 2 items covering reading comprehension from the S-TOFLA Health Literacy scale [74]. | One of the most widely used and state of the art assessments of ability to read and understand health-related materials. Tests both reading comprehension and numerical facility using health-oriented questions; low scores have been shown to be associated with lower disease knowledge in patients with chronic disease, lower use of preventative care, and higher rates of hospital admission and emergency department use. | Take low literacy levels into account, have patient repeat back in own words. |
| Numeracy |  | 2 items from the Subjective Numeracy Scale (SNS) [75] covering understanding of percentages. | The SNS effectively predicts risk comprehension without requiring participants to complete time consuming and stress-inducing math tests. | Take low numeracy levels into account, have patient repeat back in own words regular A1c checks. |
| Disease-specific items: |  |  |  |  |
|  | Diabetes | Hemoglobin a1c monitoring. | Queries whether or not the patient has had their A1c, checked at least twice per year. | 1. Make certain patient understands importance of regular A1c checks. 2. Use as teachable moment to discuss the importance of patient knowing their own and target levels. |
|  | Cardiovascular disease | Hyperlipidemia  Hypertension | Queries whether or not the patient has had their lipid levels and/or blood pressure checked at least twice per year. | Use as teachable moment to discuss the importance of patient knowing their own and target levels. |
|  | Asthma | 5 questions from the Asthma Control Questionnaire [76]. | This is **c**urrently the state-of-the-art asthma control questionnaire, developed by GlaxoSmithKline, available for free and widely used. | 1. Possibly recommend regular controller medication. 2. If on a controller, determine if asthma regimen needs to be increased. 3. For the patient – “Your asthma may not be controlled as well as it could be. Please discuss this with your doctor.” |
